# A predictive model for risk of early grade ≥ 3 infection in patients with multiple myeloma not eligible for transplant: analysis of the FIRST trial

**DOI:** 10.1038/s41375-018-0133-x

**Published:** 2018-04-26

**Authors:** Charles Dumontet, Cyrille Hulin, Meletios A. Dimopoulos, Andrew Belch, Angela Dispenzieri, Heinz Ludwig, Philippe Rodon, Jan Van Droogenbroeck, Lugui Qiu, Michele Cavo, Ann Van de Velde, Juan José Lahuerta, Olivier Allangba, Jae Hoon Lee, Eileen Boyle, Aurore Perrot, Philippe Moreau, Salomon Manier, Michel Attal, Murielle Roussel, Mohamad Mohty, Jean Yves Mary, Alexandre Civet, Bruno Costa, Antoine Tinel, Yann Gaston-Mathé, Thierry Facon

**Affiliations:** 1 Hospices Civils de Lyon, Lyon, France; 2 CHU Bordeaux, Bordeaux, France; 3 National and Kapodistrian University of Athens, Athens, Greece; 4 Cross Cancer Institute, Edmonton, AB Canada; 5 Mayo Clinic Cancer Center, Rochester, MN USA; 6 Wilhelminen Hospital, Wilhelminen Cancer Research Institute, Vienna, Austria; 7 Centre Hospitalier, Périgueux, France; 8 AZ Sint-Jan AV Brugge, Brugge, Belgium; 9 Blood Disease Hospital, Chinese Academy of Medical Science and Peking Union Medical College, Tianjin, China; 10 Seràgnoli Institute of Hematology, Bologna University School of Medicine, Bologna, Italy; 11 Universitair Ziekenhuis Antwerpen, Edegem, Belgium; 12 Hospital 12 de Octubre, Madrid, Spain; 13 Centre Hospitalier Yves Le Foll, Saint-Brieuc, France; 14 Gachon University Gil Hospital, Incheon, Korea; 15 Service des Maladies du Sang, Hôpital Claude Huriez, Lille, France; 16 CHU de Nancy, Université de Lorraine, Nancy, France; 17 University of Nantes, Nantes, France; 18 Hopitaux de Toulouse, Toulouse, France; 19 CHU Purpan/IUCT Oncopole, Toulouse, France; 20 Hôpital Saint-Antoine, Paris, France; 21 INSERM U1153, University Hospital Saint-Louis, Paris, France; 22 Quinten, Paris, France; 23 Celgene International Sàrl, Boudry, Switzerland; 24 YGM Consult, Paris, France

## Abstract

Infections are a major cause of death in patients with multiple myeloma. A post hoc analysis of the phase 3 FIRST trial was conducted to characterize treatment-emergent (TE) infections and study risk factors for TE grade ≥ 3 infection. The number of TE infections/month was highest during the first 4 months of treatment (defined as early infection). Of 1613 treated patients, 340 (21.1%) experienced TE grade ≥ 3 infections in the first 18 months and 56.2% of these patients experienced their first grade ≥ 3 infection in the first 4 months. Risk of early infection was similar regardless of treatment. Based on the analyses of data in 1378 patients through multivariate logistic regression, a predictive model of first TE grade ≥ 3 infection in the first 4 months retained Eastern Cooperative Oncology Group performance status and serum β_2_-microglobulin, lactate dehydrogenase, and hemoglobin levels to define high- and low-risk groups showing significantly different rates of infection (24.0% vs. 7.0%, respectively; *P* < 0.0001). The predictive model was validated with data from three clinical trials. This predictive model of early TE grade ≥ 3 infection may be applied in the clinical setting to guide infection monitoring and strategies for infection prevention.

## Introduction

Patients with multiple myeloma (MM) are more susceptible to infections due to advanced age, immunodeficiency caused by the underlying disease, comorbidities, and treatment toxicities [1]. Infections are a major cause of death, particularly early death, in patients with MM, highlighting the need for preventive or early treatment measures [2–6].

A scoring system can help identify patients at risk for infections during MM treatment, enabling implementation of risk-adapted strategies to prevent early infections. To identify infection risk factors, we used data from the pivotal, phase 3 FIRST trial, which compared the efficacy and safety of lenalidomide plus low-dose dexamethasone (Rd) until disease progression (Rd continuous) vs. Rd for 18 cycles (Rd18) or melphalan, prednisone, and thalidomide (MPT) in transplant-ineligible patients with newly diagnosed MM (NDMM) [7, 8].

In this post hoc analysis, a detailed characterization of infections in the FIRST trial was conducted and prognostic factors of early treatment-emergent (TE) grade ≥ 3 infections were identified. The results were used to develop a predictive model to assess the risk of this event in patients receiving standard nonintensive treatment.

## Methods

### Study design

The FIRST study (MM-020/IFM07-01; NCT00689936) has been previously reported [7]. The protocol was approved by the appropriate institutional review board or independent ethics committee before study initiation. Briefly, the multinational, open-label, randomized, phase 3 trial compared the efficacy and safety of Rd continuous vs. MPT or Rd18 in transplant-ineligible patients with NDMM. Infection prophylaxis was not mandatory in the protocol.

### Patients and assessments

Of the 1623 patients in the intent-to-treat population, TE infections were investigated in 1613 patients who received ≥ 1 treatment dose (safety population), including 532, 540, and 541 in the Rd continuous, Rd18, and MPT arms, respectively. TE infections were defined as infections occurring or worsening on or after the first dose of any study drug and up to 28 days after treatment discontinuation. Infections were identified by the investigator, classified per Medical Dictionary for Regulatory Activities and graded per Common Terminology Criteria for Adverse Events v3.0. Early infection was defined as occurring during the first 4 months of treatment. For comparison of the risk of infections between treatment arms, data from the Rd continuous and Rd18 arms were pooled (Rd pooled) and a *χ*^2^ test was used. Patients in the Rd18 and Rd continuous arms received the same treatment in the first 18 months, thereby supporting the pooling of data from these two arms for the investigation of infections in the first 4 or 18 months.

Demographics, medical history, and baseline characteristics were analyzed to identify risk factors of early TE grade ≥ 3 infection. Of 1613 treated patients, this analysis was conducted on 1378 patients (prognostic analysis population), which excluded patients who progressed, died, or discontinued treatment and had no TE grade ≥ 3 infections in the first 4 months.

External validation of the results was conducted in three independent data sets: MM-003 (NCT01311687) [9], MM-009 (NCT00056160)/MM-010 (NCT00424047) [10–12], and MM-015 (NCT00405756) [13], with 237, 444, and 391 treated patients, respectively. These trials are described in the Supplement (External Validation Trials).

The numbers of patients in the various study populations in MM-020 and the validation sets are described in Supplemental Table 1.

### Analysis of the impact of first TE grade ≥ 3 infection in the first 4 months on overall survival

A time-dependent Cox model analysis was performed to assess the impact of first TE grade ≥ 3 infection in the first 4 months on patient overall survival (OS) [14]. A multivariate analysis was conducted with all baseline prognostic factors identified in the study with the Q-Finder algorithm as described in the Supplement to assess the significance of the occurrence of first TE grade ≥ 3 infection in the first 4 months on OS, independent of the role of potential confounding factors. Results were expressed using the hazard ratio (HR) of death and its 95% CI.

### Development and validation of first TE grade ≥ 3 infection in the first 4 months risk model

Overall, 853 variables were included in an analysis to identify rules that can predict the occurrence of the first TE grade ≥ 3 infection in the first 4 months, using the Q-Finder subgroup discovery algorithm. A rule is 1 or a combination of a few variable modalities defining a group with a high or low proportion of early TE grade ≥ 3 infection. Rules were selected based on their *P*-value computed with the hypergeometric law. The statistical significance cutoff for retaining rules was determined at *P* < 5.10 × 10^−5^ to adjust for multiple testing. Twenty-five rules meeting the statistical significance threshold were retained for expert review. Additional details regarding this algorithm are provided in the Supplement (Q-Finder). Upon clinical experts’ request, the cutoff value from statistically significant rules was rounded to make it easier to use, and additional tests were performed on variables with clinical significance.

Statistically significant rules were selected by expert assessment based on their clinical and/or biological relevance to be included in a stepwise Akaike information criterion multivariate logistic regression model followed by an iterative variable selection process to remove variables with *P* ≥ 0.1 [15]. Patients with missing data on ≥ 1 input variable were excluded from the model (*n* = 9). The final model included six variables. A scoring system was developed by allocating points to factors of low (−1 or −2 points) or high risk (1 or 2 points) based on their coefficient in the multivariate logistic model. The cumulative score classified patients into high (2 to 5 points) or low (−3 to 1 points) infection risk groups. The concordance index (C-index), relative risk (RR) and its 95% CI, and number needed to treat (NNT) were determined. Assuming that a prevention treatment can reduce the risk of early TE grade ≥ 3 infection in 50% of the patients of the high-risk group, NNT is the number of patients in the high-risk group who had to receive the prevention treatment to avoid the occurrence of 1 early TE grade ≥ 3 infection. Thus, a higher NNT denotes a smaller benefit of the treatment. A *χ*^2^ test was used to compare the proportions of patients with ≥ 1 early TE grade ≥ 3 infection in the high- vs. low-risk groups. The model was tested on three independent validation data sets, and all metrics (C-index, RR, and NNT) were computed to evaluate the model.

As a confirmatory analysis (in the MM-020 and validation sets), time to first infection was estimated in the safety population using the Kaplan–Meier method in the high- and low-risk groups and the log-rank test to assess statistical significance of the difference. In addition, a competing risk analysis with progression or death without infection and infection as competing events was performed to confirm the difference in risk of first TE grade ≥ 3 infection in the first 4 months between high- and low-risk groups in the prognostic analysis population (Supplement: Competing Risk Model) [16].

## Results

### Characterization of infections

Demographic and baseline characteristics of the safety population in MM-020 are presented globally and per treatment group in Supplemental Table 2.

History of infections before enrollment was similar across treatments (Rd pooled: 27.2%; MPT: 28.5%). During the study, anti-infective drugs were prescribed to 78.5% and 67.1% of patients in the Rd pooled and MPT groups, respectively. Among the three treatment arms, 3125 infections of any grade occurred during the study; 3031 infections were TE (1.9 TE infection events per patient). Of 3031 TE infection events of any grade that occurred during the study in 1104 patients, 610 in 321 patients were grade ≥ 3 (representing 20.2% of 3025 TE infection events of known grade) (Table 1).

**Table 1:** TE infection events by grade and treatment arm in the safety population of the FIRST trial (1613 patients, including 532, 540, and 541 in the Rd continuous, Rd18, and MPT arms, respectively)

TE infection events, *n*^a^	Grade 1 (mild) infections				Grade 2 (moderate) infections				Grade 3 (severe) infections				Grade 4 (life-threatening) infections				Grade 5 (death) infections				Unknown grade infections			
	Rd cont	Rd18	MPT	Total	Rd cont	Rd18	MPT	Total	Rd cont	Rd18	MPT	Total	Rd cont	Rd18	MPT	Total	Rd cont	Rd18	MPT	Total	Rd cont	Rd18	MPT	Total
Events in the first 4 months	134	136	85	355	157	170	114	441	57	68	62	187	17	16	15	48	11	10	9	30	0	3	0	3
Events in the first 18 months	339	356	190	885	440	422	307	1169	148	145	105	398	35	27	24	86	20	20	15	55	0	3	2	5
Events beyond 18 months	175	4	4	183	174	3	1	178	62	1	0	63	6	0	0	6	2	0	0	2	1	0	0	1
Total	514	360	194	1068	614	425	308	1347	210	146	105	461	41	27	24	92	22	20	15	57	1	3	2	6

*MPT* melphalan, prednisone, and thalidomide, *Rd cont* lenalidomide plus low-dose dexamethasone until disease progression, *Rd18* lenalidomide plus low-dose dexamethasone for 18 cycles, *TE* treatment emergent

^a^A total of 79 infections occurred before the first treatment administration, and 15 infections occurred > 28 days after treatment discontinuation

During the first 18 months of treatment, 1055 patients (65.4%) and 340 patients (21.1%) experienced TE infections of any grade and TE grade ≥ 3 infections, respectively. The risk of TE infection of any grade in the first 18 months was 69.4% with Rd pooled and 57.5% with MPT (*P* < 0.0001). The risk of having ≥ 1 TE grade ≥ 3 infection during the first 18 months was 22.6% (120 patients) with Rd continuous, 22.6% (122 patients) with Rd18, and 18.1% (98 patients) with MPT (Rd pooled vs. MPT, *P* = 0.04). The risk of having a TE infection of any grade and a TE grade ≥ 3 infection beyond 18 months of treatment was 31.8% (169 patients) and 9.2% (49 patients), respectively, with Rd continuous. The risk of a TE grade 5 infection during the first 18 months was 3.6% (19 patients) with Rd continuous, 3.3% (18 patients) with Rd18, and 2.6% (14 patients) with MPT (Rd pooled vs. MPT, *P* = 0.35). After 18 months of treatment, the risk of a TE grade 5 infection was 0.4% (two patients) with Rd continuous.

### TE infections occurring during the first 4 months of treatment

**Fig. 1:**
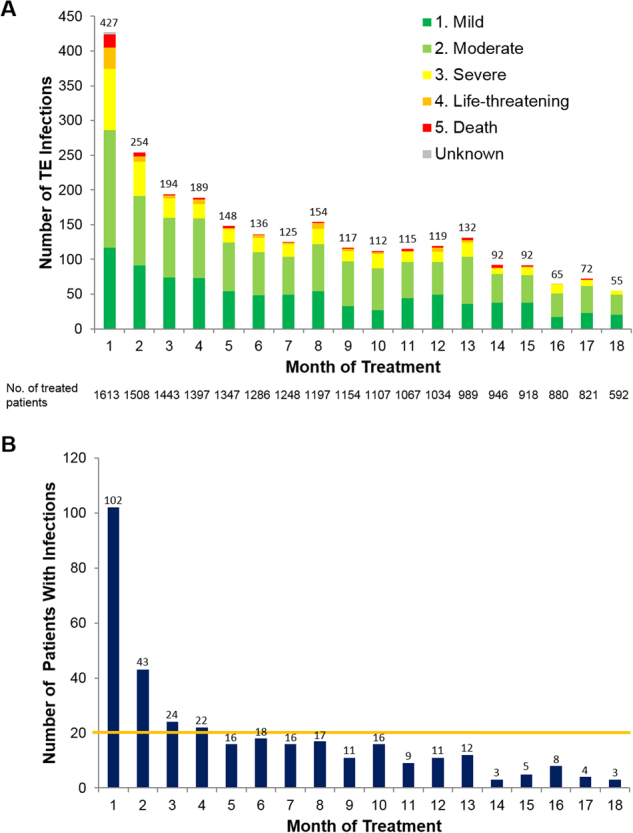
Treatment-emergent (TE) infections in the FIRST trial. **a** Number of TE infections by month in the first 18 months of the FIRST trial (1613 treated patients). The numbers above the bars indicate the total number of TE infections of all grades during the treatment month. **b** Number of new patients with TE grade ≥ 3 infections by month in the first 18 months of the FIRST trial (1613 treated patients)

The number of TE infections per month was highest during the first 4 months of treatment (Fig. 1a). A total of 1064 TE infections of any grade occurred during the first 4 months, including 265 TE grade ≥ 3 infections (representing 25.0% of 1061 TE infections of known grade) (Table 1). The lungs and respiratory tract were involved in 48.7% of early TE grade ≥ 3 infections, whereas 22.6% of these infections were localized to the blood, with patients exhibiting sepsis, bacteremia, and viremia (Supplemental Table 3). The pathogen was identified in 25.3% of early TE grade ≥ 3 infections; bacterial infections were implicated in 79.1% of cases in which a pathogen was identified (Supplemental Table 4). Streptococcal, staphylococcal, and clostridia infections were the most commonly specified bacterial infections. No statistical differences were seen between Rd pooled and MPT in the rates of staphylococcal and streptococcal infections (*P* = 0.25 and *P* = 0.15, respectively).

Overall, 56.2% of patients with a TE grade ≥ 3 infection in the first 18 months experienced their first infection in the first 4 months, and there were < 20 new patients with TE grade ≥ 3 infections per month after 4 months of treatment (Fig. 1b). A total of 191 patients (11.8%) experienced ≥ 1 TE grade ≥ 3 infection during the first 4 months of treatment (12.2% Rd pooled and 11.1% MPT, *P* = 0.51); 54 patients (3.3%) experienced > 1 TE grade ≥ 3 infection (Table 2).

**Table 2:** Rate of TE grade ≥ 3 infections by treatment arm in the FIRST trial (safety population)

Patients with indicated number of TE grade ≥ 3 infections, *n* (%)	0–4 months				0–18 months				Beyond 18 months			
	Rd cont (*n* = 532)	Rd18 (*n* = 540)	MPT (*n* = 541)	Total (*N* = 1 613)	Rd cont (*n* = 532)	Rd18 (*n* = 540)	MPT (*n* = 541)	Total (*N* = 1 613)	Rd cont (*n* = 532)	Rd18 (*n* = 540)	MPT (*n* = 541)	Total (*N* = 1 613)
0	469 (88.2)	472 (87.4)	481 (88.9)	1 422 (88.2)	412 (77.4)	418 (77.4)	443 (81.9)	1 273 (78.9)	483 (90.8)	539 (99.8)	541 (100)	1 563 (96.9)
1	46 (8.6)	48 (8.9)	43 (7.9)	137 (8.5)	72 (13.5)	72 (13.3)	64 (11.8)	208 (12.9)	35 (6.6)	1 (0.2)	0	36 (2.2)
2	13 (2.4)	16 (3.0)	8 (1.5)	37 (2.3)	28 (5.3)	35 (6.5)	22 (4.1)	85 (5.3)	10 (1.9)	0	0	10 (0.6)
≥ 3	4 (0.8)	4 (0.7)	9 (1.7)	17 (1.1)	20 (3.8)	15 (2.8)	12 (2.2)	47 (2.9)	4 (0.8)	0	0	4 (0.2)

*MPT* melphalan, prednisone, and thalidomide, *Rd cont* lenalidomide plus low-dose dexamethasone until disease progression, *Rd18* lenalidomide plus low-dose dexamethasone for 18 cycles, *TE* treatment emergent

Of the 57 TE grade five infections that occurred during the study (53 patients [3.3%]), 30 (52.6%) occurred during the first 4 months (28 patients [1.7%]).

### Impact of first TE grade ≥ 3 infection in the first 4 months on OS

The risk of death associated with a first TE grade ≥ 3 infection in the first 4 months, as assessed in a time-dependent Cox regression analysis, was significant (HR, 2.9 [95% CI, 2.4–3.6]; *P* < 0.0001). A stepwise multivariate time-dependent analysis for baseline risk factors was then performed to adjust for potential confounding factors. The occurrence of a first TE grade ≥ 3 infection in the first 4 months remained significant in the final OS predictive model (HR, 9.1 [95% CI, 5.6-14.6]; *P* < 0.0001) (Supplemental Table 5).

### Baseline factors associated with risk of ≥ 1 early TE grade ≥ 3 infection

Demographic and baseline characteristics of the intent-to-treat and prognostic analysis populations in MM-020 and the validation sets are presented in Supplemental Table 6. A comprehensive analysis was performed on the prognostic analysis population in MM-020 to identify risk factors associated with high or low risk of first TE grade ≥ 3 infection in the first 4 months using the Q-Finder algorithm (Supplemental Table 7). The most significant variables associated with a high or low risk of infection included Sβ2M levels or International Staging System stage, number of CRAB (hypercalcemia, renal failure, anemia, and bone lesions) diagnostic criteria [17], M-protein urine levels, creatinine or urea levels, red blood cell counts, hematocrit or hemoglobin levels, LDH levels, triiodothyronine (thyroid hormone; T3) levels, α-1 globulin levels, and eosinophil counts. Patients with low quality-of-life score at baseline also had a significantly increased risk of early grade ≥ 3 TE infection. An exploratory analysis of baseline immunoparesis on the risk of early grade ≥ 3 TE infection is presented in the Supplement (Immunoparesis and the Risk of Infection at 4 Months).

### First TE grade ≥ 3 infection in the first 4 months scoring system

Of the statistically significant variables identified by the Q-Finder algorithm, clinical experts in MM selected variables with high clinical relevance to be proposed to the multivariate logistic regression model (Supplemental Table 8). The multivariate analysis, which included eight rules identified by the univariate analysis to be associated with high or low risk of early TE grade ≥ 3 infection (ECOG PS < 1, ECOG PS ≥ 2, Sβ2M ≥ 6 mg/L, Sβ2M ≤ 3 mg/L, LDH ≥ 200 U/L, hemoglobin ≤ 9 g/dL, hemoglobin ≥ 11 g/dL, and creatinine ≥ 1.2 mg/dL), showed that six rules based on ECOG PS and Sβ2M, LDH, and hemoglobin levels were independently associated with first TE grade ≥ 3 infection in the first 4 months (Table 3).

**Table 3:** Multivariate logistic regression model for first TE grade ≥ 3 infection during the first 4 months of treatment (1369 patients included)

Variable	Coefficient^a^		Odds ratio	*P*-value	Points	Infection risk
	Estimate	SE				
Sβ2M ≤ 3 mg/L	−0.812	0.353	0.44	0.021	−2	Low
ECOG PS of 0	−0.403	0.216	0.67	0.062	−1	Low
Hemoglobin ≤ 11 g/dL	0.366	0.207	1.44	0.077	1	High
ECOG PS of ≥ 2	0.457	0.189	1.58	0.016	1	High
LDH ≥ 200 U/L	0.552	0.186	1.74	0.003	1	High
Sβ2M ≥ 6 mg/L	0.820	0.176	2.27	< 0.001	2	High

*ECOG PS* Eastern Cooperative Oncology Group performance status, *LDH* lactate dehydrogenase, *Sβ2M* serum β_2_-microglobulin, *TE* treatment emergent

^a^ Coefficient in the multivariate logistic model

From the resulting predictive model, a scoring system (Table 3) was used to create high (2 to 5 points) and low (−3 to 1 points) infection risk groups. The cutoff between these groups was selected based on the best sensitivity/specificity ratio. These high- and low-risk groups were associated with significantly different rates of early TE grade ≥ 3 infections (24.0% vs. 7.0%, respectively; *P* < 0.0001; C-index, 0.66; RR, 3.43 [95% CI, 2.57–4.59]; NNT, 8.3).

### Validation of the predictive model for risk of first TE grade ≥ 3 infection in the first 4 months

When tested on three independent cohorts (MM-015, MM-009/010, and MM-003), [9, 11–13] the model discriminated between high- and low-risk patients regarding the risk of developing early TE grade ≥ 3 infection (Table 4), with comparable RRs between high- and low-risk groups in all three test sets (MM-015: RR, 2.05 [*P* = 0.055]; MM-003: RR, 2.09 [*P* < 0.0001]; MM-009/010: RR, 2.09 [*P* = 0.0008]). This was despite very different populations at baseline and different rates of early TE grade ≥ 3 infection (MM-015, 9.4%; MM-009-010, 20.3%; MM-003, 43.7%) compared with MM-020 (13.9%). Due to the difference in infection risks in those populations, the NNT differed greatly in the various populations (MM-015, 15.5; MM-009/010, 5.6; MM-003, 3.2) compared with MM-020 (8.3).

**Table 4:** TE grade ≥ 3 infections during the first 4 months of high- and low-risk populations in various studies

Trial	Grade ≥ 3 infections, %		*P*-value*low risk vs. high risk	RR (95% CI)	NNT
	Low risk (−3 to 1 points)	High risk (2 to 5 points)			
MM-020 (*N* = 1 369)^a^	7.0	24.0	8.19 × 10^−19^	3.43 (2.57–4.59)	8.3
Rd pooled (*n* = 918)	7.4	24.9	2.7 × 10^−13^	3.37 (2.39–4.76)	8.0
MPT (*n* = 451)	6.2	22.4	9.15 × 10^−7^	3.63 (2.11–6.24)	8.9
MM-015 (*n* = 384)^a^	6.3	12.9	0.0552	2.05 (1.07–3.92)	15.5
MM-009/10 (*n* = 404)^a^	17.1	35.7	7.69 × 10^−4^	2.09 (1.41–3.10)	5.6
MM-003 (*n* = 222)^a^	30.3	63.3	2.21 × 10^−6^	2.09 (1.54–2.83)	3.2

*MPT* melphalan, prednisone, and thalidomide, *NNT* number needed to treat, *Rd cont* lenalidomide plus low-dose dexamethasone until disease progression, *Rd18* lenalidomide plus low-dose dexamethasone for 18 cycles, *Rd pooled* Rd cont and Rd18 patients combined, *RR* relative risk, *TE* treatment emergent

*P-value computed with *χ*^2^ test

^a^ Patients with missing data for ≥ 1 of the variables selected by the multivariate logistic regression were excluded from the high-/low-risk definition

### Confirmatory analyses of the predictive model for risk of first TE grade ≥ 3 infection in the first 4 months

**Fig. 2:**
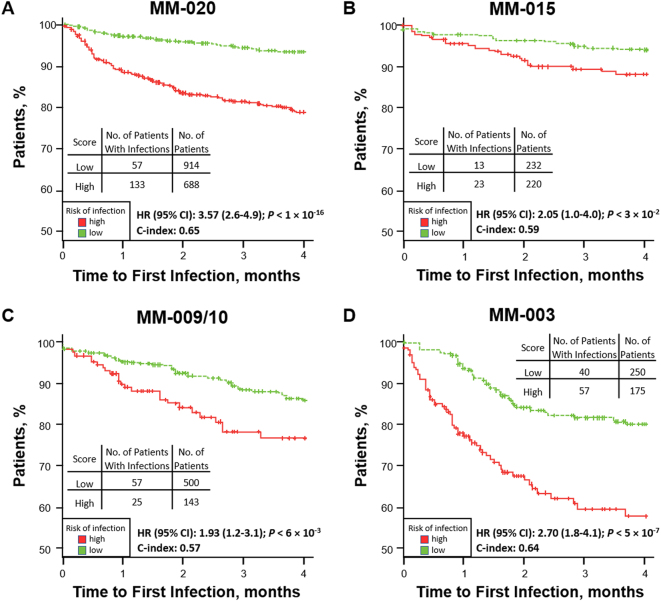
Time to first grade ≥ 3 TE infection in the first 4 months for high- and low-risk groups in the **a** MM-020 (*n* = 1602), **b** MM-015 (*n* = 452), **c** MM-009/10 (*n* = 643), **d** MM-003 (*n* = 425) populations. C-index concordance index, HR hazard ratio

For illustration, a time to first infection analysis was performed in both the MM-020 and the independent validation sets (Fig. 2). In all test sets, patients in the high-risk group had a significantly shorter time to first TE grade ≥ 3 infection in the first 4 months compared with the low-risk group (MM-020: HR, 3.6 [*P* < 0.0001], C-index, 0.65; MM-003: HR, 2.7 [*P* < 0.0001], C-index, 0.64; MM-009/010: HR, 1.9 [*P* = 0.006], C-index, 0.57; MM-015: HR, 2.05 [*P* = 0.03], C-index, 0.59).

To confirm our predictive model, a competing risks analysis with progression or death without infection as competing events with first TE grade ≥ 3 infection in the first 4 months was performed using the MM-020 data set; this analysis included the same eight rules and iterative selection process used in the multivariate logistic analysis. The competing risk analysis in MM-020 confirmed the significance of the six rules as in the logistic model (Supplemental Table 9). As such, the competing risk analysis provided an identical model to the one obtained through logistic regression analysis. The final model remained significant (*P* < 0.05) in both the MM-020 and the independent validation sets in a competing risks analysis with progression or death without infection as competing events with first TE grade ≥ 3 infection in the first 4 months.

## Discussion

Because infections remain an important cause of morbidity and mortality in patients with MM [1], analyses of large clinical trials can help identify risk factors associated with severe and life-threatening infections. The FIRST trial, which demonstrated a significant progression-free survival and OS benefit with Rd continuous vs. MPT, is among the largest phase 3 studies in MM and represents a typical transplant-ineligible NDMM population per its eligibility criteria; therefore, the prognostic factors of infection identified for these patients may be quite common in this population [7]. The FIRST trial confirmed that the risk of infection in MM is high: 65.4% of patients presented with ≥ 1 TE infection and 21.1% presented with ≥ 1 TE grade ≥ 3 infection. The risk of infection in the first 18 months was different across treatments: all TE infections (Rd pooled, 69.4%; MPT, 57.5% [*P* < .0001]) and TE grade ≥ 3 infections (Rd pooled, 22.6%; MPT, 18.1% [*P* = .04]). This was noted despite the higher rate of grade 3/4 neutropenia with MPT (44.9%) vs. Rd pooled (27.1%) [7]. Nearly 75% of all grade ≥ 3 infections occurred in the absence of neutropenia (data not shown), suggesting that dexamethasone may have a contributing role.

This post hoc analysis showed that in the first 4 months of treatment, (1) of patients who experienced a TE grade ≥ 3 infection, the majority had their first infection during this time; (2) nearly one-half of all TE grade ≥ 3 infections occurred, including the majority of infection-related deaths; and (3) first TE grade ≥ 3 infection was associated with an increased risk of death, independent of prognostic factors for OS. Our results are consistent with previous studies that have shown that infections occur more often in the first and second months of treatment [18, 19]. Infection risk may be highest during this period due to the immunosuppressive nature of active MM and antimyeloma agents coupled with the likelihood that the antimyeloma agents have not yet maximally reduced tumor load and repaired organ and tissue damage [2, 18, 20]. The risk of early TE grade ≥ 3 infection was similar with Rd vs. MPT, highlighting the role of baseline patient-specific factors in determining infection risk during early treatment.

Multivariate analysis identified ECOG PS and Sβ2M, LDH, and hemoglobin levels as prognostic factors for early TE grade ≥ 3 infection. The significance of these variables was confirmed by a competing risk analysis of first TE grade ≥ 3 infection and death or progression without infection during the first 4 months. Given that only 94 of the 3125 infections of any grade that occurred during the study were non-TE infections, it is unlikely that including non-TE infections in the analysis would alter the results. A risk-scoring system was used to separate patients in the FIRST trial into high- and low-risk groups, which were associated with significantly different rates of early TE grade ≥ 3 infections (24.0% vs. 7.0%, respectively). The predictive model differentiated high-risk from low-risk patients in three independent data cohorts, which included patients with relapsed/refractory MM (RRMM; MM-003 and MM-009/010) and NDMM (MM-015). As expected, the risk was greater in the three RRMM studies that used dexamethasone (high-dose dexamethasone in MM-009/010 and the control arm of MM-003 and low-dose dexamethasone in the pomalidomide arm of MM-003). Although still relevant, the model showed a lower absolute benefit in MM-015, which had a lower incidence of early TE grade ≥ 3 infections and used prednisone instead of dexamethasone. In the low-risk groups, the risk was similar in the MPT arms of MM-020 and MM-015, which investigated MP and MP+lenalidomide (6.2% and 6.3%, respectively). The risk was marginally higher in the Rd arms of MM-020 (7.4%) and highest in MM-009/010 and MM-003 (17.1% and 30.3 %, respectively). Similarly, RRMM studies had a significant risk of early TE grade ≥ 3 infections in the high-risk groups (up to 63.3% in the MM-003 study). Even though these findings should be interpreted cautiously, the results suggest that dexamethasone is a risk factor for early TE grade ≥ 3 infections, with studies with prednisone being associated with a lower risk.

These post hoc analysis findings are informative; however, cautious interpretation is warranted. The use of antibiotic prophylaxis was neither mandated in the study protocol nor standardized, which may limit interpretability. A pathogen could not be specified in a substantial proportion of infections reported limiting further elucidation on the types of interventions that may be useful in this setting. Although it is common in MM trials and in practice that a substantial proportion of infections have no pathogen specified [21, 22], additional MM studies with data on infections with specified causes are needed to determine possible patterns of specific types of infections and appropriate preventative therapies for patients at risk. Our model also requires further prospective interrogation for additional validation, particularly in proteasome inhibitor-based studies. Furthermore, it would be of interest for additional studies to investigate risk factors for TE grade ≥ 3 infection after the first 4 months of treatment as just over half of all TE grade ≥ 3 infections occurred after the first 4 months in this study.

In conclusion, a majority of patients in the FIRST trial reported ≥ 1 TE infection, confirming that the risk of TE infection in patients with MM is high. In addition, our analysis identified a set of baseline patient characteristics that were associated with risk of developing a TE grade ≥ 3 infection in the initial 4 months of treatment. The high- and low-risk groups defined by our scoring system were associated with significantly different infection rates, irrespective of treatment. Clinicians may be able to apply this model to adjust their monitoring and treatment strategies for infection prevention. The results of the predictive model could be integrated into current infection management guidelines, including those from the International Myeloma Working Group [23] and European Myeloma Network [24]. Future NDMM studies could apply this model to evaluate which patients (all or those at high infection risk) should receive prophylactic anti-infective drugs and what type would be most beneficial to each patient subpopulation.

## Disclaimer

The authors were fully responsible for all content and editorial decisions for this manuscript.

## Electronic supplementary material

Supplement for Dumontet MM-020 Infections MS
