# Supplementary material for: A predictive model for risk of early grade ≥ 3 infection in patients with multiple myeloma not eligible for transplant: analysis of the FIRST trial
Source: Leukemia. 2018 Apr 26;32(6):1404–13. doi: 10.1038/s41375-018-0133-x (PMC5990520; doi:10.1038/s41375-018-0133-x)
Supplement: Supplementary file 1 — Supplement for Dumontet MM-020 Infections MS [file 41375_2018_133_MOESM1_ESM.docx]

**SUPPLEMENT**

**External Validation Trials**

MM-003 (NCT01311687) was a phase 3, multicenter, open-label, randomized trial comparing pomalidomide plus low-dose dexamethasone vs high-dose dexamethasone alone for patients with RRMM who had failed ≥ 2 previous treatments of bortezomib and lenalidomide. Prophylactic antibiotic use was at the investigator’s discretion.

MM-009 (NCT00056160)/MM-010 (NCT00424047) were phase 3, multicenter, randomized, parallel-group, double-blind, placebo-controlled studies of lenalidomide plus dexamethasone vs high-dose dexamethasone alone in patients with RRMM. Routine daily antibiotic prophylaxis was recommended for all patients.

MM-015 (NCT00405756) was a phase 3, multicenter, randomized, double-blind, placebo-controlled trial that evaluated the efficacy and safety of induction therapy with MPR (melphalan + prednisone + lenalidomide) followed by lenalidomide maintenance therapy (MPR-R), compared with MPR or MP without maintenance therapy, for the treatment of patients with NDMM who were ≥ 65 years of age. Prophylactic antibiotic use was at the investigator’s discretion.

**Statistical Analysis**

Statistical analysis was performed with R software, except for the discovery of univariate predictors of high or low risk of infection, which was performed with a proprietary subgroup discovery algorithm programmed in C++ and implemented at Quinten (Paris, France; Supplement: Q-Finder).^25,26^

**Q-Finder**

Q-Finder is a proprietary supervised learning algorithm working under the following conditions:

- No particular assumption made regarding the shapes of distribution of the outcome or explanatory variables
- Outcome comprises several classes (eg, favorable/unfavorable, responder/nonresponder, dead/alive) and the algorithm explores the space of explanatory variables to identify areas of overconcentration of the class of interest specified for the exploration
- All combinations of variables (categorical and continuous) are systematically explored. Continuous variables are discretized into quantiles to control the risk of multiple testing
- The output is a set of rules. A rule is defined as a combination of variable modalities that characterizes a subgroup of patients. The variables in a rule are either continuous (discretized) variables defined by lower and upper bounds or categories for qualitative variables. The maximum number of variables per rule is generally set to 3 to keep them clinically understandable. For the sake of clinical interpretation and limitation of multiple testing, only “open” rules (rules in the form ≥ A or ≤ B) are retained
  - As an example, ECOG PS of ≥ 2 was a rule identifying a group of patients in MM-020 with a high risk of early TE grade ≥ 3 infection (24.0% experienced early TE grade ≥ 3 infections), whereas ECOG PS of 0 was a rule identifying a group of patients in MM-020 with a low risk of early TE grade ≥ 3 infection (7% experienced early TE grade ≥ 3 infections)
- Selection of rules is performed based on their size and hypergeometric *P* value. The size of a rule refers to the number of patients satisfying the rule criteria. The *P* value is defined as the probability of obtaining by chance a rule of a given size with a given proportion of the class of interest:

$$p_{hypergeometric}\left( k, r, n, N \right)=1-\sum_{i={min}_{X}}^{k-1} P\left( X=i \right)$$

for which k is the number of patients in the class of interest in the rule, r is the number of patients in the class of interest in the full data set, n is the number of patients in the rule, N is the number of patients in the full data set, min_X_ is the maximum between 0 and r + n − N, and P(X = k) is the probability of obtaining a rule defined by (k, r, n, N) as follows:

$$P\left( X=k \right)=\frac{\left( \begin{matrix} r \\ k \end{matrix} \right)\left( \begin{matrix} N-r \\ n-k \end{matrix} \right)}{\left( \begin{matrix} N \\ n \end{matrix} \right)}$$

- The rules of *P* value threshold used for selecting or eliminating rules is defined using a simple Bonferroni adjustment based on the total number of theoretical tests (total number of variables modalities × number of variables in the rule)

**Immunoparesis and the Risk of Infection at 4 Months**

As an exploratory analysis, the risk of TE grade ≥ 3 infection in the first 4 months induced by immunoparesis at baseline was also examined in common MM subtypes, including immunoglobulin G (IgG), IgA, and light chain. Low Ig levels (≤ 35% of normal value) in IgA patients (low IgG levels) and IgG patients (low IgA levels) were associated with a higher risk of TE grade ≥ 3 infection (*P* = .0037 and *P* < .001, respectively). In the overall patient population, 425 patients (30.8%) with < 40% of lower normal values of nonmonoclonal Ig had an elevated risk of TE grade ≥ 3 infection in the first 4 months (odds ratio, 2.11 [95% CI, 1.5-2.9]; *P* < .0001).

**Competing Risk Model**

A competing risk analysis of first TE grade ≥ 3 infection and death or progression without infection during the first 4 months was performed to evaluate the significance of the model:

- A competing risk regression model (in R) was used to confirm the significance of the variables (**Supplemental Table 9**).
- Output variable: occurrence of first TE grade ≥ 3 infection during the first 4 months
- Competing events: death or progression without infection
- Patients with missing data on ≥ 1 input variable among those of the model were removed: 21 patients

**Supplemental Table 1.** Study Populations (number of patients)

| **Number of patients** | **MM-020** | **MM-015** | **MM-009/010** | **MM-003** |
| --- | --- | --- | --- | --- |
| ITT population | 1 623 | 459 | 704 | 455 |
| Safety population | 1 613 | 455 | 703 | 450 |
| Excluded patients* | 235 | 64 | 259 | 213 |
| Prognostic analysis population^†^ | 1 378 | 391 | 444 | 237 |
| Patients with missing data^‡^ | 9 | 7 | 40 | 15 |
| Logistic regression population^§^ | 1 369 | 384 | 404 | 222 |
| TE grade ≥ 3 infections in the first 4 months^‖^ | 190 | 36 | 82 | 97 |

ITT, intent to treat; TE, treatment emergent.

* Patients with progression/death and no TE grade ≥ 3 infections within the first 4 months, or who discontinued treatment within the first 4 months and did not experience a TE grade ≥ 3 infection were excluded from the analysis.

**^†^** Patients included in the baseline prognostic factors analysis.

^‡^ Patients with missing data for ≥ 1 of the variables selected by the multivariate logistic regression were excluded from the high-/low-risk definition.

**^§^** Patients included in the high-/low-risk groups.

^‖^ Patients from the logistic regression population who experienced a TE grade ≥ 3 infection.

**Supplemental Table 2.** Demographic and Baseline Characteristics of the Safety Population in MM-020

| **Characteristic** | **MM-020** | | | |
| --- | --- | --- | --- | --- |
|  | **Rd Cont** | **Rd18** | **MPT** | **Total** |
|  | **(n = 532)** | **(n = 540)** | **(n = 541)** | **(n = 1 613)** |
|  |  |  |  |  |
| Age |  |  |  |  |
| Median, y | 73 | 73 | 73 | 73 |
| Range, y | 44-91 | 40-89 | 51-92 | 40-92 |
| < 70 y, n (%) | 149 (28) | 164 (30) | 157 (29) | 470 (29) |
| > 75 y, n (%) | 185 (35) | 192 (36) | 184 (34) | 561 (35) |
| Male, n (%) | 293 (55) | 273 (51) | 286 (53) | 853 (53) |
| ECOG PS, n (%) |  |  |  |  |
| 0-1 | 411 (77) | 425 (79) | 428 (79) | 1 264 (78) |
| 2-3 | 119 (22) | 115 (21) | 110 (20) | 343 (21) |
| Not available^*^ | 2 (< 1) | 0 | 3 (< 1) | 5 (< 1) |
| ISS stage, n (%) |  |  |  |  |
| I or II | 331 (62) | 334 (62) | 325 (60) | 990 (61) |
| III | 201 (38) | 206 (38) | 216 (40) | 623 (39) |
| Missing | 0 | 0 | 0 | 0 |
| MM subtype, n (%) |  |  |  |  |
| IgA | 136 (26) | 142 (26) | 122 (23) | 400 (25) |
| IgA,IgG | 7 (1) | 6 (1) | 7 (1) | 20 (1) |
| IgA,IgM | 0 | 0 | 1 (< 1) | 1 (< 1) |
| IgD | 4 (< 1) | 7 (1) | 3 (< 1) | 14 (< 1) |
| IgG | 333 (63) | 330 (61) | 348 (64) | 1011 (63) |
| IgM | 3 (< 1) | 1 (< 1) | 1 (< 1) | 5 (< 1) |
| Light Chain | 45 (8) | 53 (10) | 57 (11) | 155 (10) |
| High risk cytogenetics, n (%) | 42 (8) | 52 (10) | 47 (9) | 141 (9) |
| Missing | 285 (54) | 279 (52) | 291 (54) | 855 (53) |
| Bone lesions, n (%) | 375 (70) | 381 (71) | 386 (71) | 1 142 (71) |
| Sβ2M, n (%) |  |  |  |  |
| Median, mg/l | 4.9 | 4.7 | 4.9 | 4.8 |
| Range, mg/l | 1.3-39.4 | 1.4-44.9 | 1.5-33.9 | 1.4-44.9 |
| ≤ 3 mg/l | 104 (20) | 80 (15) | 84 (16) | 268 (17) |
| ≥ 6 mg/l | 185 (35) | 188 (35) | 207 (38) | 580 (36) |
| LDH, n (%) |  |  |  |  |
| Median, U/l | 147 | 145 | 147 | 147 |
| Range, U/l | 59-1148 | 52-2751 | 65-572 | 52-2751 |
| ≥ 200 U/l | 84 (16) | 99 (18) | 112 (21) | 295 (18) |
| Hemoglobin, n (%) |  |  |  |  |
| Median, g/dl | 10.3 | 10.4 | 10.2 | 10.3 |
| Range, g/dl | 5.6-15.9 | 5.8-16.1 | 6.1-16.8 | 5.6-16.8 |
| ≤ 11 g/dl | 356 (67) | 355 (66) | 380 (70) | 1 091 (68) |
| CrCl, n (%) |  |  |  |  |
| Median, ml/min | 61 | 60 | 61 | 61 |
| Range, ml/min | 9-186 | 4-177 | 7-198 | 4-198 |
| ≤ 60 ml/min | 269 (51) | 265 (49) | 263 (49) | 797 (49) |
| ≤ 30 ml/min | 50 (9) | 53 (10) | 57 (11) | 160 (10) |
| Albumin, n (%) |  |  |  |  |
| Median, g/dl | 3.6 | 3.6 | 3.6 | 3.6 |
| Range, g/dl | 1.6-5 | 1.6-5.2 | 1.5-5.5 | 1.5-5.5 |
| ≤ 3 g/dl | 114 (21) | 110 (20) | 128 (24) | 352 (22) |
| Neutrophils, n (%) |  |  |  |  |
| Median, GI/l | 3.0 | 3.1 | 3.0 | 3.0 |
| Range, GI/l | 0.3-16.5 | 0.7-16.0 | 0.3-11.8 | 0.3-16.5 |
| ≥ 7 GI/l | 24 (5) | 22 (4) | 17 (3) | 63 (4) |
| Platelets, n (%) |  |  |  |  |
| Median, x10^9^/L | 225 | 220 | 223 | 223 |
| Range, x10^9^/L | 44-639 | 38-768 | 46-683 | 38-768 |
| ≤ 100 x10^9^/L | 24 (5) | 27 (5) | 19 (4) | 70 (4) |
| White Blood Cells |  |  |  |  |
| Median, x10^9^/L | 5.3 | 5.3 | 5.3 | 5.3 |
| Range, x10^9^/L | 1.7-20.0 | 1.8-24.0 | 1.0-19.3 | 1.0-24.0 |
| Urea |  |  |  |  |
| Median, mg/dl | 19 | 19 | 19 | 19 |
| Range, mg/dl | 6-79 | 5-126 | 5-111 | 5-126 |
| M-Protein |  |  |  |  |
| Median, g/l | 31.2 | 31.5 | 30.7 | 31.1 |
| Range, g/l | 0-99.2 | 0-106 | 0-87.5 | 0-106 |
| High cytogenetic risk, n (%) | 42 (8) | 52 (10) | 47 (9) | 141 (9) |
| Missing | 285 (54) | 279 (52) | 291 (54) | 855 (53) |

CrCl, creatinine clearance; ECOG PS, Eastern Cooperative Oncology Group performance status; ISS, International Staging System; LDH, lactate dehydrogenase; MM, multiple myeloma; MPT, melphalan, prednisone, and thalidomide; Rd cont, lenalidomide plus low-dose dexamethasone until disease progression; Rd18, lenalidomide plus low-dose dexamethasone for 18 cycles; Sβ2M, Serum β2-microglobulin.

* Includes missing data.

**Supplemental Table 3**. Localization of TE Grade ≥ 3 Infections by Treatment Arm During the First 4 and 18 Months of Treatment in the FIRST Trial

| **Localization of TE Grade ≥ 3 Infection** | **0-4 Months** | | | **0-18 Months** | | |
| --- | --- | --- | --- | --- | --- | --- |
|  | **Rd Pooled** | **MPT** | **Total** | **Rd Pooled** | **MPT** | **Total** |
| Total number of TE grade ≥ 3 Infections, n | 179 | 86 | 265 | 395 | 144 | 539 |
| Localization, n (%) |  |  |  |  |  |  |
| Lung and respiratory tract | 83 (46.4) | 46 (53.5) | 129 (48.7) | 204 (51.6) | 77 (53.5) | 281 (52.1) |
| Sepsis and bacteremia | 48 (26.8) | 12 (14.0) | 60 (22.6) | 78 (19.7) | 23 (16.0) | 101 (18.7) |
| Gastrointestinal tract | 14 (7.8) | 7 (8.1) | 21 (7.9) | 25 (6.3) | 13 (9.0) | 35 (6.5) |
| Urinary tract | 10 (5.6) | 4 (4.7) | 14 (5.3) | 25 (6.3) | 7 (4.9) | 32 (5.9) |
| Skin | 9 (5.0) | 4 (4.7) | 13 (4.9) | 21 (5.3) | 4 (2.8) | 25 (4.6) |
| Dental and oral soft tissue | 3 (1.7) | 0 | 3 (1.1) | 5 (1.3) | 1 (0.7) | 6 (1.1) |
| Bone and joint | 1 (0.6) | 0 | 1 (0.4) | 5 (1.3) | 1 (0.7) | 6 (1.1) |
| Central nervous system | 0 | 2 (2.3) | 2 (0.8) | 2 (0.5) | 2 (1.4) | 4 (0.7) |
| Other | 2 (1.1) | 2 (2.3) | 4 (1.5) | 7 (1.8) | 4 (2.8) | 11 (2.0) |
| Not defined | 9 (5.0) | 9 (10.5) | 18 (6.8) | 23 (5.8) | 12 (8.3) | 35 (6.5) |

MPT, melphalan, prednisone, and thalidomide; Rd cont, lenalidomide plus low-dose dexamethasone until disease progression; Rd18, lenalidomide plus low-dose dexamethasone for 18 cycles; Rd pooled, Rd cont and Rd18 patients combined; TE, treatment emergent.

**Supplemental Table 4.** Microbiology of TE Infections by Treatment Arm During the First 4 and 18 Months of Treatment in the FIRST Trial

| **TE Infections, n** | **0-4 Months** | | | **0-18 Months** | | |
| --- | --- | --- | --- | --- | --- | --- |
|  | **Rd Pooled** | **MPT** | **Total** | **Rd Pooled** | **MPT** | **Total** |
| **Grade ≥ 3 Infections** | | | | | | |
| Bacterial  Clostridia  Enterococcal  *Escherichia  Klebsiella  Legionella  Neisseria*  Pseudomonal  *Salmonella*  Staphylococcal  Streptococcal  Other | 35  5  0  4  0  0  2  2  0  8  10  4 | 18  4  0  0  2  0  0  4  0  1  1  6 | 53  9  0  4  2  0  2  6  0  9  11  10 | 78  8  0  6  1  3  2  3  3  17  14  21 | 24  5  1  1  2  1  0  5  0  1  1  7 | 102  13  1  7  3  4  2  8  3  18  15  28 |
| Fungal  *Candida  Pneumocystis*  Other | 4  2  1  1 | 0  0  0  0 | 4  2  1  1 | 7  3  1  3 | 3  1  2  0 | 10  4  3  3 |
| Helminthic  Nematode | 0  0 | 0  0 | 0  0 | 1  1 | 0  0 | 1  1 |
| Protozoal  *Blastocystis* | 0  0 | 0  0 | 0  0 | 1  1 | 0  0 | 1  1 |
| Viral  Hepatitis  Herpes  Influenza  Other | 7  0  3  3  1 | 3  2  0  0  1 | 10  2  3  3  2 | 14  0  4  8  2 | 4  3  0  0  1 | 18  3  4  8  3 |
| Unspecified | **133** | **65** | **198** | 294 | 113 | 407 |
| **Total** | **179** | **86** | **265** | **395** | **144** | **539** |
| **Grade 5 Infections** |  |  |  |  |  |  |
| Bacterial  Clostridia  *Neisseria*  Pseudomonal  Staphylococcal  Streptococcal | 5  1  1  1  1  1 | 1  0  0  1  0  0 | 6  1  1  2  1  1 | 5  1  1  1  1  1 | 1  0  0  1  0  0 | 6  1  1  2  1  1 |
| Fungal  *Pneumocystis* | 0  0 | 0  0 | 0  0 | 0  0 | 1  1 | 1  1 |
| Infections—pathogen unspecified* | 16 | 7 | 23 | 34 | 12 | 46 |
| Viral  Hepatitis  Influenza | 0  0  0 | 1  1  0 | 1  1  0 | 1  0  1 | 1  1  0 | 2  1  1 |
| **Total** | **21** | **9** | **30** | **40** | **15** | **55** |

MPT, melphalan, prednisone, and thalidomide; Rd cont, lenalidomide plus low-dose dexamethasone until disease progression; Rd18, lenalidomide plus low-dose dexamethasone for 18 cycles; TE, treatment emergent.

* In total, 30 of these infections were reported as sepsis, bacteremia, viremia, and fungemia other; 13 as lower respiratory tract and lung infections; 2 as central nervous system and spinal infections; and 1 as other infections.

**Supplemental Table 5.** Stepwise Multivariate Time-Dependent Analysis for OS

| **Variable** | **Coefficient*** | | **Hazard Ratio** | ***P* value** |
| --- | --- | --- | --- | --- |
|  | **Estimate** | **SE** |  |  |
| TE grade ≥ 3 infection | 2.205 | 0.244 | 9.07 | < 2 × 10^−16^ |
| Systolic blood pressure ≤ 108 mm Hg | 0.550 | 0.165 | 1.73 | 8.60 × 10^−4^ |
| History of bone, calcium, magnesium, and phosphorus metabolism disorders^†^ = yes | 0.281 | 0.158 | 1.32 | 7.64 × 10^−2^ |
| Serum β_2_-microglobulin ≥ 8.38 mg/L | 0.352 | 0.118 | 1.42 | 2.80 × 10^−3^ |
| Renal insufficiency^‡^ = yes | −0.289 | −0.163 | 0.75 | 7.65 × 10^−2^ |
| High-risk cytogenetics**^§^** = yes | 0.702 | 0.120 | 2.02 | 4.81 × 10^−9^ |
| ECOG PS ≥ 2 | 0.332 | 0.101 | 1.39 | 9.71 × 10^−4^ |
| Fitness category combined with ISS stage^‖^ = frail with ISS stage III | −0.269 | −0.127 | 0.76 | 3.33 × 10^−2^ |
| Absolute lymphocytes ≤ 0.9 × 10^9^/L | 0.402 | 0.104 | 1.50 | 1.02 × 10^−4^ |
| ISS stage ≥ 3 | 0.474 | 0.125 | 1.61 | 1.59 × 10^−4^ |
| Red blood cells ≤ 3.1 × 10^12^/L | 0.287 | 0.088 | 1.33 | 1.16 × 10^−3^ |
| Global quality of life ≤ 50 | 0.284 | 0.092 | 1.33 | 2.05×10^−3^ |
| α-1 globulin ≥ 5% | 0.174 | 0.099 | 1.19 | 7.91 × 10^−2^ |
| Calcium (corrected) ≥ 2.54 mmol/L | 0.323 | 0.123 | 1.38 | 8.48 × 10^−3^ |
| Urea ≥ 28 mg/dL | 0.167 | 0.116 | 1.18 | 1.52 × 10^−1^ |
| Albumin (corrected) ≤ 2.9 g/dL | 0.278 | 0.119 | 1.32 | 1.94 × 10^−2^ |
| Lactate dehydrogenase ≥ 237 U/L | 0.378 | 0.136 | 1.46 | 5.53 × 10^−3^ |
| Age ≥ 78 years | 0.447 | 0.097 | 1.56 | 3.82 × 10^−6^ |
| Diastolic blood pressure ≤ 60 mm Hg | 0.221 | 0.131 | 1.25 | 9.14 × 10^−2^ |

CRAB, hyper*c*alcemia, renal failure, anemia, and bone lesions; ECOG PS, Eastern Cooperative Oncology Group performance status; ISS, International Staging System; TE, treatment emergent.

* Coefficient in the multivariate Cox model of overall survival on the whole population.

^†^ Based on high-level group term per Medical Dictionary for Regulatory Activities.

^‡^ Renal insufficiency is one of the items from the CRAB score.

**^§^** High-risk cytogenetics defined as ≥ 1 cytogenetic abnormality among t(4;14), t(14;16), and del(17p).
^‖^ Fitness category determined by the frailty score developed for the measurement of frailty based on age, Charlson Comorbidity Index score, and the activities of daily living (measures of self-care) and instrumental activities of daily living (measures of usual activities) scales from the EQ-5D questionnaire at baseline.^27^

**Supplemental Table 6.** Demographic and Baseline Characteristics of the Intent-to-Treat and Prognostic Analysis Populations in MM-020 and the Validation Sets

| **Characteristic** | **MM-020** | | **MM-015** | | **MM-009/010** | | **MM-003** | |
| --- | --- | --- | --- | --- | --- | --- | --- | --- |
|  | **ITT (n = 1 623)** | **Prognostic**  **(n = 1 378)** | **ITT**  **(n = 459)** | **Prognostic (n = 391)** | **ITT (n = 704)** | **Prognostic**  **(n = 444)** | **ITT**  **(n = 455)** | **Prognostic**  **(n = 237)** |
| Age |  |  |  |  |  |  |  |  |
| Median, y | 73 | 73 | 71 | 71 | 63 | 62 | 64 | 64 |
| Range, y | 40 - 92 | 40 - 92 | 65 - 91 | 65 - 86 | 33 - 86 | 33 - 86 | 35 - 87 | 35 - 87 |
| < 70 y, n (%) | 471 (29) | 414 (30) | 176 (38) | 156 (40) | 526 (75) | 339 (76) | 314 (69) | 163 (69) |
| > 75 y, n (%) | 567 (35) | 459 (33) | 111 (24) | 94 (24) | 68 (10) | 42 (9) | 37 (8) | 23 (10) |
| Male, n (%) | 854 (53) | 732 (53) | 228 (50) | 200 (51) | 417 (59) | 279 (63) | 268 (59) | 139 (59) |
| ECOG PS, n (%) |  |  |  |  |  |  |  |  |
| 0 | 474 (29) | 415 (30) | 71 (15) | 63 (16) | 302 (43) | 191 (43) | 146 (32) | 78 (33) |
| 1 | 795 (49) | 679 (49) | 265 (58) | 221 (57) | 317 (45) | 201 (45) | 224 (49) | 121 (51) |
| 2-3 | 349 (22) | 280 (20) | 122 (27) | 106 (27) | 71 (10) | 41 (9) | 80 (18) | 36 (15) |
| Not available | 5 (< 1) | 4 (< 1) | 1 (< 1) | 1 (< 1) | 14 (2) | 11 (2) | 5 (< 1) | 2 (< 1) |
| ISS stage, n (%) |  |  |  |  |  |  |  |  |
| I or II | 996 (61) | 870 (63) | 233 (51) | 209 (53) | 490 (70) | 320 (72) | 288 (63) | 148 (62) |
| III | 627 (39) | 508 (37) | 226 (49) | 182 (47) | 142 (20) | 77 (17) | 145 (32) | 76 (32) |
| Missing | 0 | 0 | 0 | 0 | 72 (10) | 47 (11) | 22 (5) | 13 (5) |
| Sβ2M |  |  |  |  |  |  |  |  |
| Median, mg/Dl | 4.8 | 4.7 | 5.3 | 5.1 | 3.4 | 3.2 | 4.5 | 4.5 |
| Range, mg/Dl | 1.3 - 44.9 | 1.3 - 44.9 | 1.5 - 47.2 | 1.5 - 47.2 | 1 - 45 | 1 - 45 | 1.6 - 31.8 | 1.6 - 31.8 |
| ≤ 3 mg/Dl | 268 (17) | 242 (18) | 50 (11) | 46 (12) | 294 (42) | 203 (46) | 82 (18) | 41 (17) |
| ≥ 6 mg/Dl | 580 (36) | 466 (34) | 198 (43) | 158 (40) | 116 (16) | 61 (14) | 123 (27) | 64 (27) |
| Missing | 14 (< 1) | 3 (< 1) | 1 (< 1) | 1 (< 1) | 14 (2) | 9 (2) | 20 (4) | 13 (5) |
| LDH |  |  |  |  |  |  |  |  |
| Median, U/L | 147 | 146 | 143 | 142 | 175 | 172 | 184 | 181 |
| Range, U/L | 52 - 2751 | 52 - 2751 | 55 - 416 | 55 - 416 | 77 - 765 | 82 - 611 | 70 - 2281 | 73 - 2035 |
| ≥ 200 U/L | 295 (18) | 242 (18) | 65 (14) | 48 (12) | 164 (23) | 86 (19) | 187 (41) | 91 (38) |
| Missing | 42 (3) | 2 (< 1) | 10 (2) | 6 (2) | 167 (24) | 104 (23) | 3 (< 1) | 1 (< 1) |
| Hemoglobin |  |  |  |  |  |  |  |  |
| Median, g/Dl | 10.3 | 10.4 | 10.7 | 10.8 | 11.9 | 12 | 9.9 | 9.9 |
| Range, g/Dl | 5.6 – 16.8 | 5.6 – 16.2 | 7.3 - 15.7 | 7.3 - 15.2 | 6.1 - 16 | 6.3 - 16 | 6.3 - 38 | 7.1 - 14.5 |
| ≤ 11 g/Dl | 1091 (67) | 913 (66) | 259 (56) | 213 (54) | 202 (29) | 116 (26) | 342 (75) | 180 (76) |
| Missing | 10 (< 1) | 0 | 16 (3) | 16 (4) | 167 (24) | 94 (21) | 3 (< 1) | 0 |
| Early grade 3+ infection, n (%) | 191 (12) | 191 (14) | 37 (8) | 37 (9) | 93 (13) | 93 (21) | 104 (23) | 104 (44) |
| CrCl, n (%) |  |  |  |  |  |  |  |  |
| Median, ml/min | 61 | 62 | 60.4 | 61.9 | — | — | 70 | 72 |
| Range, ml/min | 4 - 198 | 4 - 198 | 17.2 - 137.5 | 17.2 - 137.5 | — | — | 25 - 235 | 27 - 235 |
| ≤ 60 ml/min | 797 (49) | 661 (48) | 223 (49) | 180 (46) | — | — | 152 (33) | 73 (31) |
| ≤ 30 ml/min | 160 (10) | 121 (9) | 28 (6) | 18 (5) | — | — | 5 (1) | 2 (1) |
| Albumin, n (%) |  |  |  |  |  |  |  |  |
| Median, g/dl | 3.6 | 3.6 | 3.6 | 3.6 | 3.7 | 3.7 | 3.7 | 3.7 |
| Range, g/dl | 1.5 - 5.5 | 1.5 - 5.5 | 1.7 - 5.2 | 1.7 - 5.2 | 1.5 - 5.1 | 1.5 - 5 | 1.6 - 5.2 | 1.6 - 5.2 |
| ≤ 3 g/dl | 352 (22) | 285 (21) | 73 (16) | 61 (16) | 65 (9) | 29 (7) | 64 (14) | 31 (13) |
| Neutrophils, n (%) |  |  |  |  |  |  |  |  |
| Median, GI/l | 3.0 | 3.1 | 3.1 | 3.1 | — | — | 2.4 | 2.4 |
| Range, GI/l | 0.3 - 16.5 | 0.3 - 16.5 | 0.8 - 17.2 | 0.8 - 17.2 | — | — | 0.4 - 20.6 | 0.6 - 20.6 |
| ≥ 7 GI/l | 63 (4) | 55 (4) | 22 (5) | 20 (5) | — | — | 9 (2) | 3 (< 1) |
| Platelets, n (%) |  |  |  |  |  |  |  |  |
| Median, x10^9^/L | 223 | 222 | 227 | 224.5 | 208 | 212 | 133 | 137 |
| Range, x10^9^/L | 38 – 768 | 38 - 768 | 57 - 638 | 62 - 638 | 56 - 608 | 71 - 608 | 9 - 461 | 13 - 461 |
| ≤ 100 x10^9^/L | 70 (4) | 59 (4) | 9 (2) | 6 (2) | 36 (5) | 18 (4) | 142 (31) | 70 (30) |
| White Blood Cells |  |  |  |  |  |  |  |  |
| Median, , x10^9^/L | 5.3 | 5.3 | 5.4 | 5.4 | 5.1 | 5.1 | — | — |
| Range, x10^9^/L | 1.0 – 24.0 | 1.0 – 24.0 | 2.1 - 74.2 | 2.1 - 74.2 | 1.7 - 36.8 | 1.7 - 13.4 | — | — |
| Urea |  |  |  |  |  |  |  |  |
| Median, mg/dl | 19 | 19 | 19 | 18.8 | 17.1 | 17.1 | 16 | 16 |
| Range, mg/dl | 5 - 126 | 5 - 126 | 6.2 - 58 | 6.2 - 55.2 | 3.9 - 59.1 | 3.9 - 50.4 | 5 - 47 | 6 - 46 |
| High cytogenetic risk, n (%) | 142 (9) | 110 (8) | — | — | — | — | — | — |
| Missing | 861 (53) | 735 (53) | 459 (100) | — | 704 (100) | 444 (100) | 455 (100) | — |

CrCl, creatinine clearance; ECOG PS, Eastern Cooperative Oncology Group performance status; ISS, International Staging System; LDH, lactate dehydrogenase; MM, multiple myeloma; Sβ2M, Serum β2-microglobulin.

**Supplemental Table 7.** Baseline Patient Characteristics Associated With Increased or Decreased Risk of TE Grade ≥ 3 Infection in the First 4 Months Found with an Exploratory Univariate Analysis using the Q-Finder algorithm

| **Variable** | **Patients,  n (%)** | **Patients With TE Grade ≥ 3 Infections, %** | **Odds Ratio (CI)** | ***P* Value** |
| --- | --- | --- | --- | --- |
| All patients (analysis set) | 1 378 (100) | 13.9 |  |  |
| Fitness category combined with ISS stage* = frail with ISS stage III | 308 (22.4) | 22.7 | 2.31 (1.7-3.2) | 8.25 × 10^−7^ |
| ECOG PS ≥ 2^†^ | 280 (20.3) | 22.5 | 2.20 (1.6-3.1) | 5.97 × 10^−6^ |
| Serum β_2_-microglobulin ≥ 5.94 mg/L^†^ | 478 (34.7) | 24.1 | 3.43 (2.5-4.7) | 6.66 × 10^−15^ |
| Serum β_2_-microglobulin ≤ 2.89 mg/L^†^ | 207 (15.0) | 3.4 | 0.19 (0.1-0.4) | 7.79 × 10^−8^ |
| M-protein urine ≥ 616.1 mg/24 h | 376 (27.3) | 20.7 | 2.06 (1.5-2.8) | 8.33 × 10^−6^ |
| Urea ≥ 23 mg/dL | 452 (32.8) | 21.0 | 2.30 (1.7-3.1) | 1.24 × 10^−7^ |
| Urea ≤ 19 mg/dL | 731 (53.0) | 9.8 | 0.48 (0.4-0.7) | 3.26 × 10^−6^ |
| α-1 globulin (corrected) ≥ 0.4 g/dL | 624 (45.3) | 18.9 | 2.18 (1.6-3.0) | 6.10 × 10^−7^ |
| Creatinine ≥ 1.2 mg/dL^†^ | 445 (32.3) | 20.4 | 2.14 (1.6-2.9) | 1.36 × 10^−6^ |
| Lactate dehydrogenase ≥ 200 U/L^†^ | 242 (17.6) | 24.0 | 2.38 (1.7-3.4) | 1.79 × 10^−6^ |
| T3 (thyroid hormone) ≤ 268 pg/dL | 469 (34.0) | 19.8 | 2.05 (1.5-2.8) | 4.61 × 10^−6^ |
| Red blood cells ≤ 2.86 × 10^12^/L | 276 (20.0) | 23.2 | 2.32 (1.7-3.2) | 1.48 × 10^−6^ |
| Red blood cells ≥ 3.51 × 10^12^/L | 522 (37.9) | 7.7 | 0.39 (0.3-0.6) | 5.74 × 10^−8^ |
| Hematocrit ≤ 27.5% | 293 (21.3) | 22.5 | 2.23 (1.6-3.1) | 2.98 × 10^−6^ |
| ­Hematocrit ≥ 35.6% | 276 (20.0) | 6.2 | 0.35 (0.2-0.6) | 6.88 × 10^−6^ |
| Hemoglobin ≤ 9.4 g/dL^†^ | 367 (26.6) | 21.0 | 2.09 (1.5-2.9) | 6.12 × 10^−6^ |
| Hemoglobin ≥ 10.787 g/dL^†^ | 555 (40.3) | 8.5 | 0.44 (0.3-0.6) | 8.00 × 10^−7^ |
| ISS stage > 2 | 508 (36.9) | 21.5 | 2.63 (1.9-3.6) | 7.43 × 10^−10^ |
| CRAB score ≥ 2 | 378 (27.4) | 21.7 | 2.26 (1.7-3.1) | 4.52 × 10^−7^ |
| Eosinophils ≥ 2.01% | 563 (40.9) | 8.7 | 0.45 (0.3-0.6) | 1.82 × 10^−6^ |
| QoL scores (EORTC QLQ-C30) at baseline^‡^ |  |  |  |  |
| Physical functioning ≥ 58.333 | 824 (59.8) | 9.8 | 0.44 (0.3-0.6) | 1.31 × 10^−7^ |
| Side effects ≥ 23.333 | 436 (31.6) | 20.0 | 2.01 (1.5-2.7) | 9.81 × 10^−6^ |
| Side effects ≤ 11.111 | 543 (39.4) | 8.3 | 0.43 (0.3-0.6) | 5.22 × 10^−7^ |
| Mobility ≤ 1 | 587 (42.6) | 9.0 | 0.47 (0.3-0.7) | 3.80 × 10^−6^ |
| Global QOL ≥ 66.667 | 485 (35.2) | 8.5 | 0.46 (0.3-0.7) | 7.34 × 10^−6^ |

ECOG PS, Eastern Cooperative Oncology Group performance status; EORTC, European Organisation for Research and Treatment of Cancer; ISS, International Staging System; QoL, quality of life; T3, triiodothyronine; TE, treatment emergent.

**^*^** Fitness category determined by the frailty score developed for the measurement of frailty based on age, Charlson Comorbidity Index score, and the activities of daily living (measures of self-care) and instrumental activities of daily living (measures of usual activities) scales from the EQ-5D questionnaire at baseline.^27^

^†^ Hypergeometric variable included in the multivariate model.

^‡^ Mobility, physical functioning, side effects, and global QoL are items from the EORTC QLQ-C30.

**Supplemental Table 8.** Baseline Patient Characteristics Associated With Increased or Decreased Risk of TE Grade ≥ 3 Infection in the First 4 Months Found by Univariate Analysis Requested by Clinical Experts

| **Variable** | **Patients,  n (%)** | **Patients With TE Grade ≥ 3 Infections, %** | **Odds Ratio (CI)** | ***P* Value** |
| --- | --- | --- | --- | --- |
| All patients (analysis set) | 1 378 (100) | 13.9 |  |  |
| Serum β_2_-microglobulin ≥ 6 mg/L^†^ | 466 (33.8) | 24.2 | 3.42 (2.5-4.7) | 8.10 × 10^−15^ |
| Serum β_2_-microglobulin ≤ 3 mg/L ^†^ | 242 (17.6) | 4.1 | 0.23 (0.1-0.4) | 7.11 × 10^−8^ |
| Creatinine ≥ 1.2 mg/dL ^†^ | 445 (32.3) | 20.4 | 2.14 (1.6-2.9) | 1.36 × 10^−6^ |
| Lactate dehydrogenase ≥ 200 U/L^†^ | 242 (17.6) | 24.0 | 2.38 (1.7-3.4) | 1.79 × 10^−6^ |
| Hemoglobin ≤ 9 g/dL^†^ | 256 (18.6) | 22.7 | 2.18 (1.5-3.1) | 1.34 × 10^−5^ |
| Hemoglobin ≥ 11 g/dL^†^ | 496 (36.0) | 8.3 | 0.44 (0.3-0.6) | 2.43 × 10^−6^ |
| ISS stage < 2 | 290 (21.0) | 6.6 | 0.37 (0.2-0.6) | 1.17 × 10^−5^ |
| ECOG PS < 1^†^ | 415 (30.1) | 8.2 | 0.46 (0.3-0.7) | 2.38 × 10^−5^ |
| ECOG PS ≥ 2^†^ | 280 (20.3) | 22.5 | 2.20 (1.6-3.1) | 5.97 × 10^−6^ |
| Albumin ≤ 3 g/dL | 285 (20.7) | 20.4 | 1.84 (1.3-2.6) | 4.14 × 10^−4^ |
| Platelets ≤ 100 × 10^9^/L | 59 (4.3) | 28.8 | 2.66 (1.5-4.8) | 1.67 × 10^−3^ |
| Absolute lymphocytes ≤ 1 × 10^9^/L | 292 (21.2) | 17.8 | 1.48 (1.0-2.1) | 1.95 × 10^−2^ |
| Absolute neutrophils ≥ 7 × 10^9^/L | 55 (4.0) | 29.1 | 2.69 (1.5-4.9) | 2.05 × 10^−3^ |
| Age ≥ 65 to ≤ 70 years | 410 (29.8) | 13.4 | 0.95 (0.7-1.3) | 4.14 × 10^−1^ |
| Age ≥ 75 years | 548 (39.8) | 14.6 | 1.11 (0.8-1.5) | 2.85 × 10^−1^ |
| Female | 646 (46.9) | 13.8 | 0.99 (0.7-1.3) | 4.98 × 10^−1^ |
| Occurrence of an infection in the month preceding the first treatment intake = yes | 112 (8.1) | 26.8 | 2.51 (1.6-3.9) | 1.16 × 10^−4^ |

CRAB, hyper*c*alcemia, renal failure, anemia, and bone lesions; ECOG PS, Eastern Cooperative Oncology Group performance status; EORTC, European Organisation for Research and Treatment of Cancer; ISS, International Staging System; TE, treatment emergent.

^†^ Variable included in the multivariate model.

**Supplemental Table 9.** Competing Risk Model for first TE Grade ≥ 3 Infection During the First 4 Months of Treatment as Compared to the Logistic Regression Model

| Variable | **Competing Risk (n = 1602)** | | | **Logistic Regression (n = 1369)** |
| --- | --- | --- | --- | --- |
|  | **Coefficient** | | ***P* Value** | **Coefficient**  **Estimate** |
|  | **Estimate** | **SE** |  |  |
| ECOG PS = 0 | −0.427 | 0.210 | .042 | -0.403 |
| ECOG PS ≥ 2 | 0.360 | 0.165 | .029 | 0.457 |
| Hemoglobin ≤ 11 g/dL | 0.389 | 0.197 | .048 | 0.366 |
| LDH ≥ 200 U/L | 0.330 | 0.169 | .051 | 0.552 |
| Sβ2M ≤ 3 mg/L | −0.817 | 0.356 | .022 | -0.812 |
| Sβ2M ≥ 6 mg/L | 0.669 | 0.167 | 6 × 10^−5^ | 0.820 |

ECOG PS, Eastern Cooperative Oncology Group performance status; LDH, lactate dehydrogenase; Sβ2M, serum β_2_-microglobulin; TE, treatment emergent.
